# Diagnostic stewardship aiming at expectorated or induced sputum promotes microbial diagnosis in community-acquired pneumonia

**DOI:** 10.1186/s12879-022-07199-4

**Published:** 2022-03-02

**Authors:** Bjørn Waagsbø, Eva Margrethe Buset, Jørn-Åge Longva, Merete Bjerke, Birgitte Bakkene, Anne-Stine Ertesvåg, Hanne Holmen, Marko Nikodojevic, To Thy Tran, Andreas Christensen, Einar Nilsen, Jan Kristian Damås, Lars Heggelund

**Affiliations:** 1 Regional Centre for Disease Control in Central Norway Regional Health Authority, St. Olavs Hospital Trondheim University Hospital, Trondheim, Norway; 2 Central Norway Hospital Pharmacy Trust, Ålesund, Norway; 3 Department of Medicine, Ålesund Hospital, Møre and Romsdal Hospital Trust, Ålesund, Norway; 4 Central Norway Hospital Pharmacy Trust, Trondheim, Norway; 5 Department of Microbiology, St. Olavs Hospital, Trondheim University Hospital, Trondheim, Norway; 6 Department of Clinical and Molecular Medicine, Norwegian University of Science and Technology, Trondheim, Norway; 7 Department of Microbiology, Møre and Romsdal Hospital Trust, Ålesund, Norway; 8 Department of Infectious Diseases, St. Olavs Hospital, Trondheim University Hospital, Trondheim, Norway; 9 Department of Clinical and Molecular Medicine, Centre of Molecular Inflammation Research, NTNU, Trondheim, Norway; 10 Department of Clinical Science, Faculty of Medicine, University of Bergen, Bergen, Norway; 11 Department of Internal Medicine, Drammen Hospital, Vestre Viken Hospital Trust, Drammen, Norway

**Keywords:** Pneumonia, Community-acquired pneumonia, Aetiology, Microbiology, Diagnostic yield, Expectorated sputum, Induced sputum, Antibiotic stewardship, Diagnostic stewardship

## Abstract

**Purpose:**

Studies on aetiology of community-acquired pneumonia (CAP) vary in terms of microbial sampling methods, anatomical locations, and laboratory analyses, since no gold standard exists. In this large, multicentre, retrospective, regional study from Norway, our primary objective was to report the results of a strategic diagnostic stewardship intervention, targeting diagnostic yield from lower respiratory tract sampling. The secondary objective was to report hospitalized CAP aetiology and the diagnostic yield of various anatomical sampling locations.

**Methods:**

Medical records from cases diagnosed with hospitalized CAP were collected retrospectively from March throughout May for three consecutive years at six hospitals. Between year one and two, we launched a diagnostic stewardship intervention at the emergency room level for the university teaching hospital only. The intervention was multifaceted aiming at upscaling specimen collection and enhancing collection techniques. Year one at the interventional hospital and every year at the five other emergency hospitals were used for comparison.

**Results:**

Of the 1280 included cases of hospitalized CAP, a microbiological diagnosis was established for 29.1% among 1128 blood cultures and 1444 respiratory tract specimens. Blood cultures were positive for a pathogenic respiratory tract microbe in 4.9% of samples, whereas upper and lower respiratory tract samples overall provided a probable microbiological diagnosis in 21.3% and 47.5%, respectively. Expectorated or induced sputum overall provided aetiology in 51.7% of the samples. At the interventional hospital, the number of expectorated or induced sputum samples were significantly increased, and diagnostic yield from expectorated or induced sputum was significantly enhanced from 41.2 to 62.0% after the intervention (p = 0.049). There was an over-representation of samples from the interventional hospital during the study period. Non-typeable *Haemophilus influenza* and *Streptococcus pneumoniae* accounted for 25.3% and 24.7% of microbiologically confirmed cases, respectively.

**Conclusion:**

Expectorated or induced sputum outperformed other sampling methods in providing a reliable microbiological diagnosis for hospitalized CAP. A diagnostic stewardship intervention significantly improved diagnostic yield of lower respiratory tract sampling.

**Supplementary Information:**

The online version contains supplementary material available at 10.1186/s12879-022-07199-4.

## Introduction

Pneumonia is the most prevalent infectious disease that leads to hospitalization [1]. Pathogenesis encompasses the transmission of infectious microbes to the respiratory epithelium, and subsequently micro-aspirations to the alveoli [2]. Over decades *Streptococcus pneumoniae* has invariantly been reported as the most frequent pathogen in CAP, but in later years non-typeable *Haemophilus influenza* (NTHi) has emerged as most frequent in some studies [3, 4].

Microbiological diagnosis of lower respiratory tract infections remains challenging. High quality studies with targeted protocols have provided only poor or medium quality results, even with the incorporation of novel technologies or invasive techniques to detect the infecting agent [5]. Also, patient characteristics vary greatly in both age, acquisition, aetiology, severity, systemic involvement, immune response, and coexisting diseases. Ultimately, it is challenging to design and conduct rigorous studies on pneumonia that give meaningful and generalizable results.

In Scandinavian countries, empiric antimicrobial therapy have traditionally relied on narrow-spectrum beta-lactams, such as penicillin V and G, primarily aiming at the traditionally most important bacterial CAP microbe in a Nordic setting, *Streptococcus pneumoniae* [6, 7].

A specific pathogen-directed antimicrobial therapy is endorsed by most professional societies [1], although a majority of pneumonias will succumb to empirical therapy alone and without efforts to secure a microbiological diagnosis. Recommendations for empiric antimicrobial therapy therefore still have to rely on the composite of patient characteristics, travel history, exposure to known transmission settings, disease severity, and the knowledge of local prevalence of antimicrobial resistance.

Underscored by the ongoing covid-19 pandemic, there is accelerating recognition of viruses as causes of pneumonia. Furthermore, other layers of uncertainty and complexity in the microbiological diagnosis of pneumonia is connected to the role of colonizing or true infectious microbes, and the clinical implications of polymicrobial infections [8, 9].

In this study, we summarized the results on the microbiological diagnosis of hospitalized CAP, in a regional, low antimicrobial resistance prevalence setting. In addition, we wanted to explore the potential of diagnostic stewardship measures to achieve enhanced diagnostic yield of expectorated or induced sputum sampling for microbiological diagnosis.

## Materials and methods

Patients hospitalized with the diagnosis of pneumonia (ICD-10 J13-J18.9) in the county of Møre and Romsdal, and Sør-Trøndelag in the period March–May for three consecutive years from 2016 to 2018, were considered for inclusion. These months were chosen in order to minimalize the impact of influenza virus disease, and restructuring of hospital staffing during the summer holiday. Five local hospitals and a larger teaching university hospital cover nearly 580.000 inhabitants in the region.

Prerequisites for inclusion were a minimum of 24-h hospital stay in the medical, pulmonary or intensive care unit, initiation of antibiotics for pneumonia, and the pneumonia diagnosis reported as a primary diagnosis. Persons under 18 years of age, nosocomial pneumonias, CAP complicated with secondary nosocomial infections during the treatment course, readmissions within 30 days, as well as pneumonia as secondary diagnosis, were excluded.

**Fig. 1:**
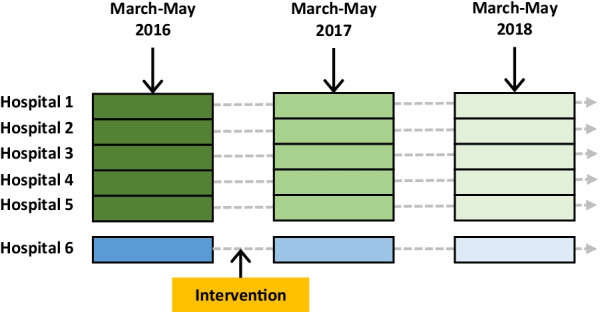
Inclusion periods at the six hospitals and the point of intervention at hospital 6 along a time axis

Over the inclusion period, diagnostic stewardship measures targeting specimen sampling for microbiological confirmation were implemented. The intervention was launched subsequently to the first study year in 2016 at hospital 6 only. The two ensuing post-intervention years were compared with the pre-intervention year. The five other hospitals were used for comparison. Figure 1 describes the inclusion periods and timing of the intervention.

Included in the intervention was the publication of charts indicating diagnostic yield of the respiratory specimens from various anatomical locations, review lectures to emergency department staff, including on call doctors and nurses, and specific training sessions emphasizing correct timing, motivational factors and expectoration techniques, as depicted by the European Respiratory Society at their online resource centre [10]. All interventional measures aimed at upscaling specimen collection numbers, both expectorated and induced sputum, and enhancing health care provider and patient techniques. Microbiologists at the laboratories were unaware of the intervention, seeking to maintain standard testing strategy during post-intervention study years. Detailed information about the intervention is available in the Additional file 1: Appendix.

A retrospective data collection was performed after each inclusion period. Discharge letters, medical records, radiological journal, and laboratory data from microbiological and biochemistry tests were recorded. In cases difficult to interpret, a joint study task force consisting of specialists in infectious diseases and clinical microbiology reviewed the patient information.

Bacteriological confirmation was established with the use of conventional laboratory culture techniques. Detection of pathogenic bacteria in blood cultures were consistently considered significant. Respiratory tract pathogens were considered significant if respiratory tract samples yielded mono- or duo-microbial growth in semi-quantitative cultures. Lower respiratory tract samples were cultured if microscopy by the microbiologist revealed significant leucocytosis, as outlined by the polymorphonuclear to squamous epithelial cell ratio [11]. Atypical pathogens and respiratory viruses were detected by the use of nuclear acid amplification techniques (NAAT). The decision to perform NAAT was based on both requests from the attending doctor as well as individual clinical evaluation by the microbiologist at the study sites. All three independent and collaborating, public microbiological laboratories in the region performed all analyses. Protocols to aid decisions were largely identical between laboratories.

Diagnostic yield from microbiological testing strategies were defined as the proportions of samples with a detectable, reliable pathological airway microbe that were targeted by antimicrobial therapy, as compared to all patients undergoing microbiological testing. The study group used all the collected data to determine the clinical role of the detected pathogen. Appointed variables were presented with simple descriptive statistics. Microbiological data from the five local hospitals were compared to the corresponding data from the university hospital. We applied the chi-square test for binominal data to detect statistical differences in sampling numbers and diagnostic yield between hospitals and years in the study period.

The protocol for each study year was evaluated and approved by the Regional Ethics Committee (2017/1439), data protection officials, and hospital administrations for both health trusts.

## Results

The study identified 1852 unique hospital stays for CAP, of which 1280 (69%) met all inclusion criteria. Of these 63% and 37% were admitted to the five local hospitals combined and the university hospital, respectively. Microbiological analyses were performed at three laboratories. Descriptive statistics for the whole patient population, the clinical, laboratory and imaging data are presented in Table 1.

**Table 1:** Patient and selected diagnostic and infection characteristics in included CAP cases

Variable	Hospital 1	Hospital 2	Hospital 3	Hospital 4	Hospital 5	Hospital 6	All
Intervention site	No	No	No	No	No	Yes	
n	123	283	132	117	158	467	1280
Age average(years)	71.5	75.1	71.8	71.7	73.6	69.8	72
Age > 65 years (%)	74.8%	81.2%	75.0%	69.2%	75.3%	69.8%	73.9%
Male gender (%)	58.1%	50.1%	58.6%	50.4%	49.4%	52.3%	52.1%
Nursing home resident (%)	4.1%	3.5%	8.3%	1.7%	5.1%	5.8%	4.9%
Comorbidity status							
Chronic obstructive pulmonary disease	13 (10.6)	32 (11.3)	16 (12.1)	23 (19.7)	31 (19.6)	61 (13.1)	176 (13.8)
Chronic congestive heart disease	10 (8.1)	38 (13.4)	18 (13.6)	20 (17.1)	36 (22.8)	58 (12.4)	180 (14.1)
CRB65 score (%)							
0	22.0%	12.0%	22.0%	19.7%	22.2%	25.1%	20.7%
1	46.3%	50.5%	41.7%	50.4%	55.7%	46.0%	48.2%
2	24.4%	29.0%	28.8%	23.1%	17.1%	23.1%	24.4%
3	7.3%	8.1%	7.6%	6.0%	5.1%	5.1%	6.3%
4	0.0%	0.4%	0.0%	0.9%	0.0%	0.6%	0.4%
Antimicrobial therapy before microbiological testing (average per year)	23.4%	19.4%	17.4%	23.4%	19.4%	20.6%	20.6%
ICU admittance, n (%)	19 (15.4)	42 (14.8)	26 (19.7)	23 (19.7)	10 (6.3)	37 (7.9)	157 (12.2)
Positive pressure ventilation							
Non-invasive, n (%)	9 (7.3)	34 (12.0)	12 (9.1)	14 (12.0)	13 (8.2)	87 (18.6)	169 (13.2)
Invasive, n (%)	1 (0.8)	4 (1.4)	5 (3.8)	4 (3.4)	2 (1.2)	5 (1.1)	21 (1.6)
Definite or probable new radiological infiltrate, n (%)	87 (70.7)	251 (88.7)	101 (76.5)	98 (83.4)	143 (90.5)	418 (89.5)	1098 (85.6)
Diagnostic tests performed							
Nasal secretions, n (%)	23 (18.7)	78 (27.6)	27 (20.5)	23 (19.7)	41 (25.9)	112 (24.0)	304 (23.8)
Pharyngeal secretions, n (%)	11 (8.9)	14 (4.9)	14 (10.6)	11 (9.4)	17 (10.8)	54 (11.6)	121 (9.5)
Expectorated or induced sputum, n (%)	35 (28.5)	65 (23.0)	34 (25.8)	34 (29.1)	60 (38.0)	171 (36.6)	399 (31.2)
Tracheal secretions, n (%)	1 (0.8)	4 (1.4)	5 (3.8)	4 (3.4)	2 (1.3)	5 (1.3)	21 (1.6)
Bronchoalveolar lavage, n (%)	2 (1.6)	6 (2.1)	2 (1.5)	2 (1.7)	3 (1.9)	11 (2.4)	26 (2.0)
Pleural effusion aspiration, n (%)	1 (0.8)	3 (1.1)	1 (0.8)	1 (0.9)	2 (1.3)	16 (3.4)	24 (1.9)
Blood culture, n (%)	118 (95.9)	272 (96.1)	121 (91.7)	112 (95.7)	139 (88.0)	452 (96.8)	1214 (94.8)
NAAT, n (%)	26 (21.1)	68 (24.0)	34 (25.8)	22 (18.8)	44 (27.8)	127 (27.2)	321 (25.1)

*NAAT* nuclear acid amplification test

**Fig. 2:**
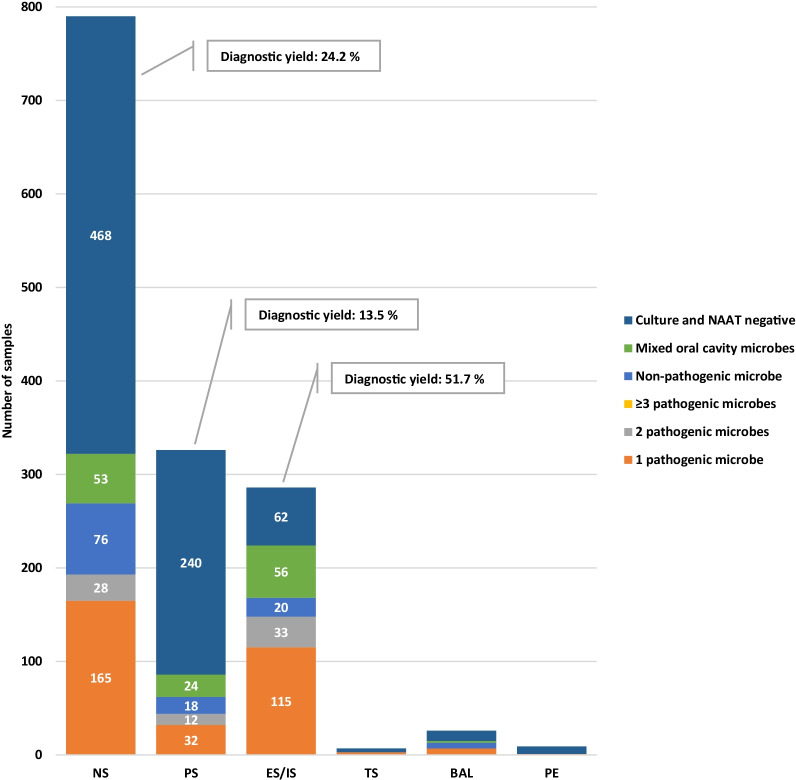
Numbers of respiratory tract samples (in absolute numbers on bars) and corresponding diagnostic yield from microbiological testing from various anatomical sampling sites among all hospitals and years. *NS* nasopharyngeal secretions, *PS* pharyngeal secretions, *ES/IS* expectorated or induced sputum, *TS* tracheal secretions, *BAL* bronchoalveolar lavage, *PE* pleural effusion

Nasopharyngeal, pharyngeal, tracheal, and bronchoalveolar secretions, expectorated or induced sputum, and aspirated pleural effusion were not routinely or consistently collected among patients ascribed with pneumonia diagnosis. In addition, disease severity correlated inconsistently with microbiological sampling. For all years and hospitals combined, a total of 1.444 respiratory tract samples were subjected for microbiological analyses, of which non-pathogenic airway microbes were reported in 120 (8.3%) tests, and mixed oral cavity flora in 135 (9.3%) tests. For 793 of 1444 (54.9%) patient samples, laboratory testing reported a negative result. In 908 of 1280 cases (70.9%), the pneumonia diagnosis was established without respiratory tract specimen sampling. Samples collected from the various anatomical locations are presented in Table 1, and the distributions of polymicrobial infections are presented in Fig. 2.

The diagnostic yield of respiratory tract samples collected from different anatomical locations demonstrated considerable variations in performance. Expectorated or induced sputum from all years and hospitals combined, conferred microbiological confirmation in 148 of 286 (51.7%) cases, whereas the corresponding yield for nasopharyngeal, pharyngeal, and tracheal secretions, bronchoalveolar lavage and pleural effusion was 24.2%, 13.5%, 42.9%, 26.4% and 11.1% respectively.

**Fig. 3:**
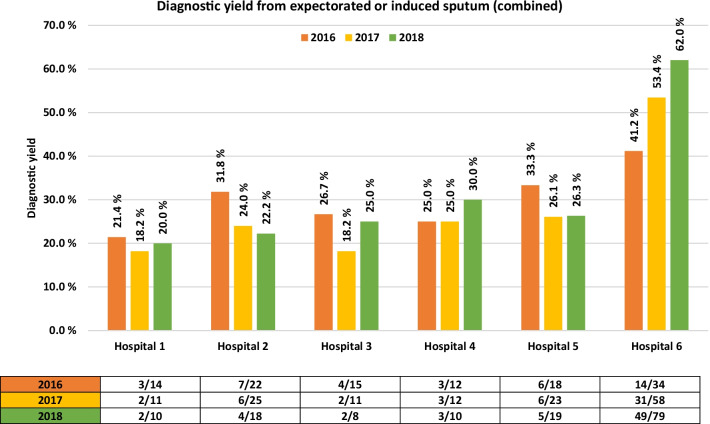
Change in diagnostic yield from expectorated or induced sputum between hospitals and pre- and post-interventional years

Specific diagnostic stewardship measures were systematically implemented in the university hospital between the two first study years from 2016 to 2017 (Fig. 1). There was a statistically significant increase in the number of patients undergoing expectorated or induced sputum collection, *Χ*^*2*^ (5, N = 425) = Chi-square statistic value 9.8705, p = 0.007, in this period. Also, a significantly higher diagnostic yield from expectorated or induced sputum samples was demonstrated between 2016 and 2018, *Χ*^*2*^ (3, N = 114) = Chi-square statistic value 3.8888, p = 0.04861. By the end of the study, diagnostic yield from expectorated or induced sputum reached 62.0%. There were no statistically significant differences between hospitals in the pre-interventional year, but samples from the interventional hospital were over-represented during the study period. Results are presented in Fig. 3.

For the entire cohort, aetiological diagnosis was established in 372 of 1280 (29.1%) of the pneumonia cases. The infecting agent was evident by blood cultures in 55 of 1128 (4.9%) samples. In upper respiratory tract specimens, cultures and nucleic acid amplification test (NAAT), yielded 197 of 1116 (17.7%) aetiological confirmations in mono-microbial infections, and 40 of 1116 (3.6%) in duo-microbial infections. Corresponding results from lower respiratory tract specimens were 126 of 328 (38.4%) in mono-microbial infections and 30 of 328 (9.1%) in duo-microbial infections. Nucleic acid amplification test (NAAT) detected the infecting pathological agent in 46 of 286 (16.1%) of the lower airway samples.

**Fig. 4:**
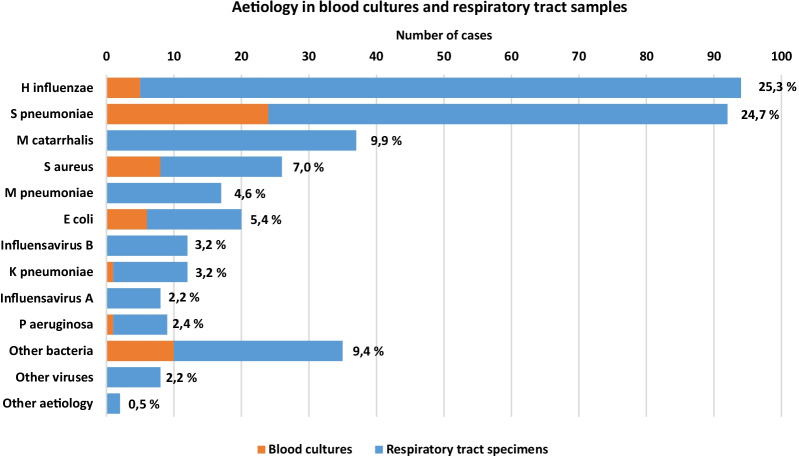
Frequencies of microbiological aetiology in blood cultures and upper and lower respiratory tract samples

NTHi and *Streptococcus pneumoniae* were the most frequently isolated microbes in blood cultures and respiratory tract specimens combined, accounting for 94 (25.3%) and 92 (24.7%) of 372 microbiologically confirmed pneumoniae cases respectively. Respiratory viruses were detected in 28 of 372 (7.5%) microbiologically confirmed pneumonia cases. No pathogenic microbes harbouring special drug-resistant phenotypic patterns were recovered, such as extended spectrum beta-lactamase (ESBL), methicillin-resistant *Staphylococcus aureus* (MRSA), or multidrug resistant *Pseudomonas aeruginosa*. *Streptococcus pneumoniae* was uniformly penicillin-susceptible. NTHi in blood cultures and lower respiratory tract samples were ampicillin resistant in 20.0% and 19.1%, respectively. Aetiological findings from respiratory tract samples and blood cultures are presented in Fig. 4.

## Discussion

This study provides further insight to the aetiological diagnosis and distributions of hospitalized CAP in a low antibiotic resistance prevalence setting. Aetiology was confirmed by routine microbiological testing in 29.1% of included cases. In addition, strategic diagnostic stewardship measures demonstrated that efforts to target microbiological sampling frequencies and techniques turned out successful, in terms of enhanced microbiological diagnostic yield.

The proportions of patients that routinely underwent procedures to collect samples from the respiratory tract were comparable to previous studies [3, 9, 12–14]. A diagnostic yield of 4.9% in blood cultures and 39.5% in cultured respiratory tract samples is also within the range found in comparable studies. It is noteworthy that a particularly high overall diagnostic yield of 51.7% was observed in expectorated or induced sputum, and this outperformed other anatomical sampling sites.

Samples from the upper respiratory tract are not routinely used for determining CAP aetiology. However, such samples were frequently performed to included cases in our study. A previously published review on the usefulness of aetiological tests for CAP concluded that samples from the upper respiratory tract should be performed to patients that are unable to produce an adequate, purulent sputum at admission [15]. This was also reflected in Swedish clinical practice guidelines from 2016 [16].

Expectorated or induced sputum is the standardized procedure for tuberculosis management. However, it’s role in common lower respiratory tract infections has faded over time, although approximately 75% of patients can produce an adequate sputum sample at admission [17], and that sensitivity of sputum examination is > 75% for detecting bacterial pathogens [18]. In our study, expectorated or induced sputum provided considerable diagnostic yield of 51.7%, although this diagnostic strategy was only applied to 31.2% of patients diagnosed with CAP. Furthermore, by relatively modest interventional efforts, the diagnostic yield of expectorated or induced sputum increased to 62.0%. We are not aware of similar results from interventions aiming at enhancing diagnostic yield from expectorated or induced sputum at a ward-level in CAP. It seems that such samples nonetheless provide valuable microbiological confirmations in CAP and should be the preferred method for respiratory tract sampling. However, sampling from the respiratory tract is inevitable hampered by infection control measures in viral pandemic situations. Our study was underpowered to detect whether diagnostic yield from lower respiratory tract samples were benefitted from increased numbers or quality of expectorated or induced sputum. Also of note, bronchoalveolar lavage sampling was infrequent, most likely due to low numbers of severe disease, low AMR prevalence, and few complications. For this reason, we concluded that the calculated diagnostic yield did not reflect expected yield.

NTHi as the most prevalent CAP pathogen in our study, is in line with recent studies from Denmark [3] and Germany [19]. Traditionally, community-acquired lower respiratory tract infections in patients with structural pulmonary diseases, especially chronic obstructive pulmonary disease (COPD), are more likely to be caused by NTHi [4, 20]. Of notice, we found COPD in only 27% of patients with NTHi infection. This may indicate that clinical practice guidelines in Nordic countries underestimate the prevalence of NTHi infections in CAP, and thereby offer inadequate therapy recommendations. The potential emerging relative prevalence of NTHi in CAP, may be related to pneumococcal vaccination, although an absolute increase is also possible [21]. In Norway, people aged > 65 years, or diagnosed with conditions known to increase risk of pneumococcal disease, are recommended to receive a pneumococcal vaccine, but data on adherence are lacking. In addition, routine pneumococcal vaccination to children was introduced in 2006.

No clinical signs or symptoms in acute respiratory tracts infections are pathogen specific. International guidelines on diagnostic strategies and antimicrobial therapy in hospitalized CAP often favour thorough microbiological evaluation and testing, in particular in severe infections [22]. Even so, exposure to special transmission settings, underlying comorbid conditions, and disease severity all represent considerable pitfalls to microbiological testing and empiric antimicrobial therapy outcomes. In our study, the lack of a consistent diagnostic testing strategy was evident at the study sites.

A recent review claims that representative respiratory tract secretions applied to highly sensitive nucleic acid amplification tests (NAAT) today have the capacity to detect common viral and bacterial pathogens as well as selected drug-resistant determinants [23]. Turnaround time for NAAT tests targeting multiple viral and bacterial pathogens are increasingly rapid and may decline to minutes. In terms of antimicrobial stewardship, a negative test may withhold empirical coverage, and a positive test may permit individualized pathogen-directed therapy. Further, efforts to establish a reliable microbiological diagnosis in pneumonia have proved beneficial in terms of clinical outcomes and resource utilization. Both mortality [24], overall antimicrobial therapy consumption [25], broad spectrum antibiotic consumption [26], infection-control practices [27], and length of stay [28], are significantly reduced by such strategy. Our study was conducted with the use of traditional cultures of respiratory tract secretions. NAAT provided aetiological confirmation in only 16% of tests in our study.

The diagnostic yield of any strategy to detect the infecting bacteria in CAP is likely to be influenced by the timing of specimen collection in view of antimicrobial therapy. In our study, 20.6% of included cases received antimicrobial therapy before microbiological sampling. A rigorous study of CAP among immune-competent adults, demonstrated that the infecting agent was significantly more frequently detected in blood cultures prior to empirical antimicrobial therapy [12]. The same finding did not apply for respiratory tract specimens. International guidelines have previously stated that pre-treatment Gram stain and culture of expectorated sputum should be performed only if good-quality specimens can be obtained and quality performances measures for collection, transport, and processing of samples can be met [29]. In a recent published systematic review, Gram staining of sputum samples still seem to provide valuable diagnostic information, in particular for *S. pneumonia* and *H. Influenzae* detections, in an antibiotic stewardship perspective [30].

Severity assessment in pneumonia is not routinely conducted and documented in clinical practice, especially outside of intensive care settings. The CRB65-score is uniformly recommended to aid empirical antimicrobial therapy in all settings, and to assess microbiological diagnostic strategies [7]. With few exceptions, the study group calculated the CRB65-score retrospectively in our study. This may indicate that other undocumented approaches, if any, to assess disease severity, exist. In our cohort, the distributions of CRB65-score of 1 or 2 was 69–77%, and CRB65-score of 3–4 was 4–10% among all study sites. These findings indicate that included cases were largely non-severe CAP, and that disease severity did not differ significantly between study sites. It also indicates that the hospitalization for non-severe CAP is common, contrary to guideline recommendations, and that other circumstances for hospitalization are often emphasized.

Antimicrobial stewardship measures are considered crucial to prevent harmful outcomes from antimicrobial resistance [31]. In countries with low AMR prevalence, microbiological confirmed cases of CAP allow for pathogen-directed, narrow-spectrum therapy. Of importance, only 29% of included hospitalizations for CAP cases underwent microbiological diagnostic approach in our study. This should encourage clinicians to reinforce sampling techniques and to scale up sampling numbers, preferably lower respiratory tract secretions.

Testing for respiratory viruses in a broad panel scale is encouraged by antibiotic stewardship guidelines to reduce inappropriate antimicrobial usage [32]. This recommendation relies on studies that have classical pre- and post-intervention models, to calculate the reduction of antibiotic consumption. Other strategies, combining NAAT testing with serum biomarkers or host immune-response analyses, shows promising results [33]. We did not undertake antibiotic usage calculations in the present study. Moreover, we wanted to describe the aetiology of hospitalized CAP in a region with low prevalence of antimicrobial resistance, and to highlight that diagnostic yield from lower respiratory tract specimens may increase with the use of simple efforts to sustain adequate sampling.

The study has some limitations. Firstly, all data from included cases were extracted retrospectively. Secondly, inclusion criteria relied on the attending doctor’s ability to correctly catalogue patient data. Thirdly, we may have missed designated respiratory tract specimens collected in primary health care settings prior to hospitalization. Fourthly, details on the individual patient’s ability to comply with testing strategy recommendations were not available. Fifthly, respiratory tract samples were stored overnight, and for three hospitals transported to the laboratory before handled. Finally, microbiology results may be affiliated by the non-identical in-house laboratory protocols and procedures among the laboratories.

In conclusion, this study shows that modest efforts to scale up sampling frequencies and enhance sampling techniques, provided significantly more microbiological confirmations in hospitalized CAP. Also, expectorated or induced sputum outperformed other respiratory secretions. We advise others to conduct similar interventions in order to establish rigorous cost–benefit analyses for the role of such interventions, and to calculate the potential reduction of antimicrobial consumption. We also emphasize the need for closer adherence to clinical practice guidelines in terms of diagnostic approaches.

## Supplementary Information

**Additional file 1.** Appendix: Detailed information about the intervention.

## Data Availability

The data generated are available from the corresponding author on reasonable request.
