# Supplementary material for: Diagnostic stewardship aiming at expectorated or induced sputum promotes microbial diagnosis in community-acquired pneumonia
Source: BMC Infect Dis. 2022 Mar 2;22:203. doi: 10.1186/s12879-022-07199-4 (PMC8889388; doi:10.1186/s12879-022-07199-4)
Supplement: Supplementary file 1 — Additional file 1. Appendix: Detailed information about the intervention. [file 12879_2022_7199_MOESM1_ESM.pdf]

Appendix

Intervention to enhance diagnostic yield from expectorated or induced sputum

Setting: Emergency room at a university teaching hospital in Norway

Patient selection: In-hospital community-acquired pneumonia

Model: Classical pre- and post-intervention strategy

Intervention: Multifaceted intervention within the ER at a large university teaching hospital based on the following measures:

1) Publication of charts to employees (including on call medical doctors and nurses).
    a. Diagnostic yield of nasopharyngeal samples in upper/lower respiratory tract infections.
    b. Diagnostic yield of pharyngeal samples in upper/lower respiratory tract infections.
    c. Diagnostic yield of expectorated or induced sputum samples in lower respiratory tract infections.
2) Review lectures to employees in the emergency room
    a. 4 sessions (15 – 25 employees at each time), 30 minutes per time.
    b. Content:
        i. Various infections in the lower respiratory tract.
        ii. Various patient categories with infections in the lower respiratory tract.
        iii. Indications for microbiological sampling.
        iv. Methodological aspects of microbiological sampling
            1. Hospital care provider's preparations
            2. Patient preparations
            3. Utilities to perform sampling
            4. Expectorational techniques
            5. Induced sputum techniques
            6. Transportation of samples to the laboratory
3) Training sessions
    a. 4 sessions (15 – 25 employees at each time), 30 minutes per time.
    b. On call doctors and nurses in the emergency room.
    c. Sessions mostly performed with authentic patients.
    d. Content:
        i. Determining indication for sampling.
        ii. Preparations.
        iii. Sampling techniques.
        iv. Demonstration of expectorate production.

v. Demonstration of induced sputum procedure.

Authors comment

Point 2 and 3 were largely based on both in-hospital clinical practice procedures and online information from the European Respiratory Society website; E-learning resources.
